# Intestinal Mucosal Immunity-Mediated Modulation of the Gut Microbiome by Oral Delivery of *Enterococcus faecium* Against *Salmonella Enteritidis* Pathogenesis in a Laying Hen Model

**DOI:** 10.3389/fimmu.2022.853954

**Published:** 2022-03-15

**Authors:** Shimeng Huang, Xiaoping Rong, Meiling Liu, Zhongjun Liang, Yanqiang Geng, Xinyue Wang, Jianyun Zhang, Cheng Ji, Lihong Zhao, Qiugang Ma

**Affiliations:** ^1^ State Key Laboratory of Animal Nutrition, College of Animal Science and Technology, China Agricultural University, Beijing, China; ^2^ Poultry Mineral Nutrition Laboratory, College of Animal Science and Technology, Yangzhou University, Yangzhou, China

**Keywords:** *E. faecium*, hens, *S. Enteritidis*, intestinal health, gut microbiota, performance

## Abstract

*Enterococcus faecium* (*E. faecium*) is a protective role that has crucial beneficial functions on intestinal homeostasis. This study aimed to investigate the effects of *E. faecium* on the laying performance, egg quality, host metabolism, intestinal mucosal immunity, and gut microbiota of laying hens under the *Salmonella Enteritidis* (*S. Enteritidis*) challenge. A total of 400 45-week-old laying hens were randomly divided into four treatments (CON, EF, SCON, and SEF groups) with five replicates for each group and 20 hens per replicate and fed with a basal diet or a basal diet supplemented with *E. faecium* (2.5 × 10^8^ cfu/g feed). The experiment comprised two phases, consisting of the pre-salmonella challenged phase (from day 14 to day 21) and the post-salmonella challenged phase (from day 21 to day 42). At day 21 and day 22, the hens in SCON and SEF groups were orally challenged with 1.0 ml suspension of 10^9^ cfu/ml *S. Enteritidis* (CVCC3377) daily, whereas the hens in CON and EF groups received the same volume of sterile PBS. Herein, our results showed that *E. faecium* administration significantly improved egg production and shell thickness during salmonella infection. Also, *E. faecium* affected host lipid metabolism parameters *via* downregulating the concentration of serum triglycerides, inhibited oxidative stress, and enhanced immune functions by downregulating the level of serum malondialdehyde and upregulating the level of serum immunoglobulin G. Of note, *E. faecium* supplementation dramatically alleviated intestinal villi structure injury and crypt atrophy, and improved intestinal mucosal barrier injuries caused by *S. Enteritidis* challenge. Moreover, our data revealed that *E. faecium* supplementation ameliorated *S. Enteritidis* infection-induced gut microbial dysbiosis by altering the gut microbial composition (reducing *Bacteroides*, *Desulfovibrio*, *Synergistes*, and *Sutterella*, and increasing *Barnesiella*, *Butyricimonas*, *Bilophila*, and *Candidatus_Soleaferrea*), and modulating the gut microbial function, such as cysteine and methionine metabolism, pyruvate metabolism, fatty acid metabolism, tryptophan metabolism, salmonella infection, and the PI3K-Akt signaling pathway. Taken together, *E. faecium* has a strong capacity to inhibit the *S. Enteritidis* colonization of hens. The results highlight the potential of *E. faecium* supplementation as a dietary supplement to combat *S. Enteritidis* infection in animal production and to promote food safety.

## Introduction

*Salmonella enterica* serovar Enteritidis (*S. enterica*) infection is an important public health problem and also associated with significant morbidity and mortality in those infected with the pathogen (1, 2). Intestinal damage and diarrhea caused by *S. enterica* infection are a severe gastrointestinal disease responsible for the annual economic loss in the global poultry industry (3). Salmonella infection is associated with intestinal damage, impaired absorption of nutrients, and poor overall performance in poultry. Also, salmonella is one of the most common reasons of foodborne disease (4–6). Salmonella, as a common foodborne pathogen that affects humans, poses a serious threat to human health *via* contaminated poultry products, including laying eggs and meat (6). It was well known that the risks of acquiring this disease are greatly influenced by the prevalence of salmonella in poultry (7). Also, infection with salmonella significantly increases chick mortality and disrupts egg formation to reduce laying performance and egg quality in hens (8, 9). Very importantly, the stages of the laying period of laying hens are generally 27–50 weeks old. Egg production and egg quality by the hens in the peak egg production stage are affected by manifold factors, especially salmonella infection (10, 11). Besides, salmonella infection in hens also disrupts host metabolism and intestinal barrier functions and promotes host inflammatory responses and oxidative stress. Hence, to prevent the salmonella contamination of poultry products worldwide, growing studies demonstrated that feed additives are also considered as effective measures to minimize salmonella infection in poultry production (12, 13). In recent years, antibiotic use is the main approach to confront pathogenic infection; however, due to the emergence of antibiotic-resistant bacteria in animal husbandry (14, 15), there is an urgent need to find suitable alternatives to improve the intestinal health and maintain animal and human health in the post-antibiotics era (2, 16). Many studies have demonstrated that probiotic supplementation might be an effective strategy to solve this public problem in poultry (17, 18).

The current definition formulated by the Food and Agriculture Organization of the United Nations and World Health Organization working group experts states that probiotics are “live strains of strictly selected microorganisms which, when administered in adequate amounts, confer a health benefit on the host” (19, 20). Therefore, the use of probiotics is a promising measure for the prevention and treatment of intestinal disorders or diseases, such as inflammatory bowel disease (IBD), diarrhea, and intestinal homeostasis dysfunction (21–23). Numerous studies have also shown that some microbes, including *Bacillus*, *Lactobacilli*, and *Enterococci*, could significantly alleviate the severity and damage of intestinal ecosystems in poultry (24–26). *Enterococcus faecium* (*E. faecium*) is a ubiquitous bacterium that has been observed in the microbiota of many animals and humans (27). Also, the commensal strains of *E. faecium* have been reported to protect animals from enteric pathogenic infection and potentially improve host metabolism, immune responses, and intestinal homeostasis (27, 28). Studies have shown that the chicken supplement *E. faecium* could significantly improve the growth performance of broiler chickens and promote the digestion and absorption of nutrients (29). Also, another study revealed that *E. faecium* NCIMB 10415 has inhibited the pathogenic infection in piglets (30). For example, *E. faecium* has a considerable therapeutic effect on *S. typhimurium* infection in pigs (31). Moreover, current studies have demonstrated that the supplementation of *E. faecium* NCIMB11181 can decrease salmonella colonization in the intestine of broiler chickens infected with *Escherichia coli* O78 (32). While some previous studies have begun to reveal the functions of *E. faecium* in animal husbandry, the underlying mechanisms of action and specific protective factors for *E. faecium* that are related with salmonella susceptibility in laying hens are still unknown.

Therefore, our study mainly explored the detailed protective mechanism of *E. faecium* against *S. enterica* infection in laying hens, from the sides of crucial phenotypes, host metabolism and immune responses, intestinal barrier function, gut microbial community and structure, and salmonella colonization.

## Materials and Methods

The experiment was carried out following the guidelines of China Agricultural Animal Care and Use Ethics Committee and conducted according to the relevant guidelines and regulations (AW02211202-1-1, Beijing, China). All experimental protocols were in accordance with the recommendations of the Guide for Guidelines for Experimental Animals of the Ministry of Science and Technology (Beijing, China).

### Animals, Housing, and Experimental Design

**Figure 1:**
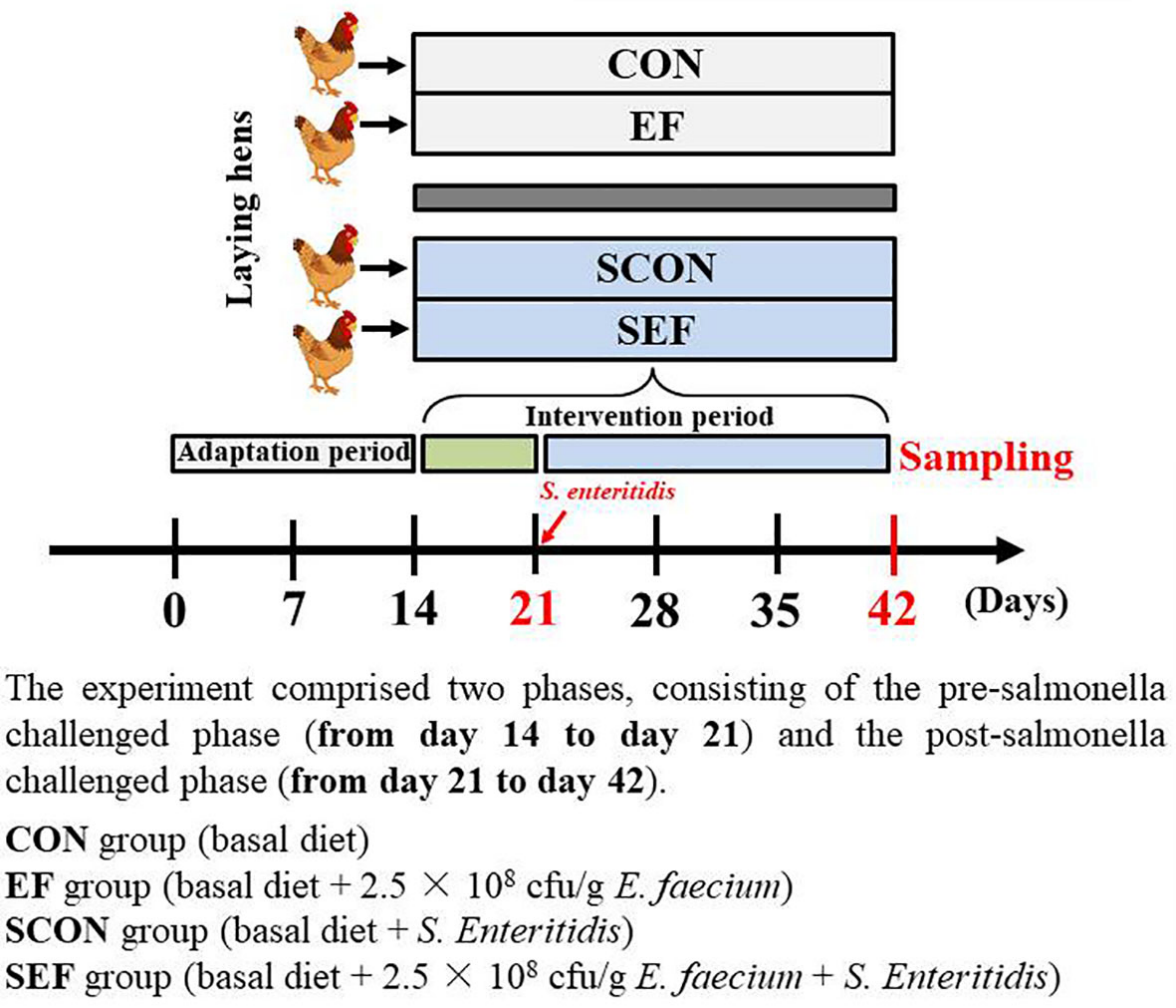
Study design for the whole experiment.

A total of 400 45-week-old laying hens (Yukou Poultry Co., Ltd., Beijing, China) were assigned according to the body weight (1.80 ± 0.10 kg) and egg production (88 ± 2.10%) and then randomly allocated to four groups with five replicates for each group and 20 hens per replicate. Each of the groups consisted of five replicates in ten different cages (two birds per cage). The cages (Height 45 cm × Width 45 cm × Depth 45 cm) were equipped with one nipple drinker and an exterior feed trough that expanded the length of the cage. The whole experiment comprised 6 weeks, consisting of a 2-week adaptation period and 4- week experimental period. As shown in **Figure 1**, the experiment comprised two phases, consisting of the pre-salmonella challenged phase (from day 14 to day 21) and the post-salmonella challenged phase (from day 21 to day 42). The *E. faecium* powder (2.5 × 10^8^ cfu/g feed) used in this study was provided by a commercial company (Smistyle Sci. & Tech. Development Co., Ltd, Beijing, China). The laying hens were fed with a basal diet (powder) or a basal diet supplemented with *E. faecium* (2.5 × 10^8^ cfu/g feed). The four treatment groups were as follows: CON group (basal diet), EF group (basal diet + 2.5 × 10^8^ cfu *E. faecium* per g feed), SCON group (basal diet + *S. Enteritidis*), and SEF group (basal diet + 2.5 × 10^8^ cfu *E. faecium* per g feed *+ S. Enteritidis*). In our study, the required quality of probiotics was added to the commercial powdered feed for homogenous mixing. Also, the basal diet was formulated to meet the recommended nutrient content by the National Research Council (1994) and was shown in **Table S1**. Room temperature was thermostatically controlled at 22 ± 3°C, with 16 h of light/d. The laying hens were provided with *ad libitum* feed and water throughout the experiment. In our study, challenged and non-challenged hens were fed in two independent, mechanically ventilated hen houses under the same environmental conditions to avoid cross-infection. Before and during the experiment, the salmonella- free status of diets, water, and environment samples was determined by using RT-qPCR analysis as we have discussed previously (33).

### *S. Enteritidis* Inoculum and Challenge

*S. enterica* spp. *enterica serovar* Enteritidis (*S. Enteritidis*, CVCC3377) was obtained from the China Institute of Veterinary Drug Control (Beijing, China). The frozen culture was recovered by using 10 ml of sterile tryptone soy broth and incubated at 37°C with orbital shaking for 24 h. Subsequently, 5 ml of *S. Enteritidis* pre-culture were transferred to 100 ml of tryptone soy broth and incubated with orbital shaking overnight at 37°C. To determine the concentration of viable *S. Enteritidis* in the culture, the inoculum was diluted with sterile phosphate buffer saline (PBS) (pH = 7.2), then plated on xylose lysine doxycholate (XLD) and incubated at 37°C for 24 h. The stock culture was prepared in sterile PBS and adjusted to 1 ×10^9^ cfu/ml of *S. Enteritidis* to be used as an inoculum. At day 8 and day 9, the hens in SCON and SEF groups were orally challenged with 1.0 ml suspension of 10^9^ cfu/ml *S. Enteritidis* daily, whereas the hens in CON and EF groups received the same volume of sterile PBS. A syringe with an attached flexible tube was used for the administration of the suspension and the physiological saline solution.

### Laying Performance and Egg Quality

During the study, feed intake was recorded weekly by calculating the difference between full bucket weights and the remaining feed. The hen-day egg production and egg weight were recorded daily, and the body weight was recorded weekly on a replication basis. The egg mass was calculated. The feed conversion ratio (FCR) was calculated as the grams of feed intake per gram of egg mass produced. At the end of the experiment, 30 eggs from each treatment were randomly collected to assess egg quality parameters. The eggshell strength and eggshell thickness were measured with a digital egg tester (ESTG-01; Orka Food Technology Ltd., Ramat Hasharon, Israel). The Haugh unit, yolk color, and egg weight were measured by a multifunctional egg quality tester (EA-01; Orka Food Technology Ltd., Ramat Hasharon, Israel). The eggshell was weighed, and yolks were separated using a separator and were then weighed to determine the relative yolk and albumen proportion as previously described (34).

### Blood Sampling and Biochemical Analysis

At the end of the experiment, blood samples were collected from birds *via* the wing vein on sampling days as previously described. The serum was centrifuged at 3,000 rpm for 15 min at room temperature. Serum samples were aspirated by a pipette and stored in 1.5 ml tubes at -20°C until analyzed. The serum concentrations of total protein (TP), albumin (ALB), globulin (GLB), alanine aminotransferase (ALT), aspartate aminotransferase (AST), alkaline phosphatase (ALP), high-density lipoprotein cholesterol (HDL-C), low-density lipoprotein cholesterol (LDL-C), total cholesterol (TC), triglycerides (TGs), calcium (Ca), phosphate (P), immunoglobulin A (IgA), immunoglobulin G (IgG), and immunoglobulin M (IgM) were measured by an automatic biochemical analyzer (7600; Hitachi, Tokyo, Japan), following the manufacturer’s instructions. Also, the levels of total superoxide dismutase (T-SOD), total antioxidant capacity (T-AOC), malondialdehyde (MDA), interleukin-1β (IL-1β), interleukin-6 (IL-6), interferon-γ (IFN-γ), and tumor necrosis factor-α (TNF-α) were evaluated with enzyme-linked immunosorbent assay kits (Nanjing Jiancheng Biology Engineering Institute, Nanjing, China). All operations are in accordance with the instructions. Hens were humanely euthanized using an injection of pentobarbital sodium (0.4 ml kg/body weight; Sile Biological Technology Co. Ltd., Guangzhou City, China).

### Tissue Collection, Fixation, and Histochemistry

At the end of the experiment, duodenum, jejunum, and ileum tissues were fixed in 4% paraformaldehyde, embedded in paraffin, cut into 5 μm thick sections, and subsequently stained with hematoxylin and eosin (H&E). Images were collected and analyzed using the CaseViewer 2.4 software (3DHISTECH Ltd., Budapest, Hungary). The villus height (VH) was measured from the top of the villus to the crypt mouth, and the crypt depth (CD) was defined as the depth of the invagination between adjacent crypt mouths. The villus width was measured at the bottom of the villus. Intestinal tissue damage was scored as previously described (35, 36), and the epithelial loss of intestinal villi and the infiltration of inflammatory cells were evaluated.

### Immunofluorescence Staining and Analysis

Paraffin-embedded 5 μm thick sections were deparaffinized by heating to 60°C for 15 min and cleared with xylene, followed by an ethanol gradient (75%, 95%, and 100%) and water and steamed for 30 min in a citrate buffer for antigen retrieval. The levels of apoptosis in the colon tissues were detected by terminal deoxynucleotidyl transferase (TdT) dUTP nick-end labeling (TUNEL) staining according to the instructions provided as previously described (Roche, Basel, Switzerland). The 4’,6-diamidino-2-phenylindole, dilactate (DAPI) blue nuclei with the same label were selected as the total cells, and the TUNEL-positive cell number per field of intestinal epithelial cells was analyzed. Cell apoptosis was observed by green fluorescence microscopy (×200 magnification).

The intestinal tissues, including duodenum, jejunum, and ileum, harvested from hens were fixed in 4% PFA in PBS and incubated in 50% ethanol overnight. After fixation, the tissues were embedded in paraffin, sectioned, and subjected. For immunofluorescence analysis, samples were incubated with a ZO-1 rabbit polyclonal antibody (Cat: #A0659, ABclonal Technology; 1:200) and occludin (OCLN) rabbit polyclonal antibody (Cat: # A2601, ABclonal Technology; 1:200) for 30 min at 37°C. After three washes, the samples were stained with a Cy3-conjugated goat anti-rabbit secondary antibody (Beyotime, Beijing, China) and DAPI (Cat: #D21490, Invitrogen Life Technologies, Carlsbad, CA, USA). Images were obtained using a fluorescence microscope (Carl Zeiss, Oberkochen, Germany).

### Cecal DNA Extraction, Amplification, and Sequencing of 16s RNA Gene

All the fresh cecal contents were collected and immediately frozen in liquid nitrogen and instantly sent to the laboratory for DNA extraction. Microbial DNA was isolated by the Omega Bio-Tek stool DNA kit (Omega, Norcross, GA, United States) and quantified by a NanoDrop 2000 spectrophotometer (Thermo Fisher Scientific, Waltham, MA, USA). Then, the V3–V4 region of the 16S rRNA gene was amplified with 338F and 806R primers with the sequence of 5’-ACTCCTACGGGAGCAGCA-3’ and 5’-GGACTACHVGGGTWTCTAAT-3’. DNA samples were quantified, followed by the amplification of the V3–V4 hypervariable region of the 16S rDNA. The final amplicon pool was evaluated by the AxyPrep DNA gel extraction kit. The purified PCR products were sequenced on the Illumina MiSeq PE300 platform at Major Biomedical Technology Co., Ltd. (Shanghai, China).

After demultiplexing, the resulting sequences were merged with FLASH (v1.2.11) and quality-filtered with fastp (0.19.6). Then, the high-quality sequences were de-noised using the DADA2 plugin in the QIIME2 (version 2020.2) [81] pipeline with the recommended parameters, which obtains single-nucleotide resolution based on the error profiles within samples. DADA2-denoised sequences are usually called amplicon sequence variants (ASVs). To minimize the effects of sequencing depth on the alpha and beta diversity measure, the number of sequences from each sample was rarefied to 4,000, which still yielded an average Good’s coverage of 97.90%. The taxonomic assignment of ASVs was performed using the naive Bayes consensus taxonomy classifier implemented in QIIME2 and the SILVA 16S rRNA database (v138). The analyses of the 16S rRNA microbiome sequencing data were performed using the free online platform Majorbio Cloud Platform.

### Determination Gene Copy Numbers of *S. Enteritidis* in Cecal Contents

Five randomly chosen hens from SCON and SEF groups were euthanized, respectively. The samples of fresh cecal contents were aseptically collected and immediately frozen. The genomic DNA from samples was isolated with the QIAamp DNA Stool Mini Kit (Qiagen GmbH, Hilden, Germany) according to the manufacturer’s protocols. Finally, the genomic DNA of *S. Enteritidis* in these samples was determined in 0.1 μl aliquot of the DNA template for RT-qPCR detection.

The sequences of the primer pairs used for RT-qPCR detection were designed according to the *S. Enteritidis* special Prot6E nucleotide sequence (NO. U66901). The production size was 175 bp. The sequences of the primer pairs were as follows: Prot6E-F: 5’-ACAGGGGCACAATAACCGTA-3’ and Prot6E-R: 5’-TGCATCCCTGTCACAACATT-3’. The PCR and data acquisition and analysis were performed using the iCycler iQ Optical system software (version 3.1; Bio-Rad, California, USA). The number of target copies in the reaction was deduced from the threshold cycle (Ct) values. The Ct value corresponds to the fractional cycle number at which the fluorescence emission exceeds the standard deviation of the mean baseline emission by 15-fold. The plasmid DNA containing the target amplicon was diluted to contain 10^1–^10^8^ copies of the target DNA per test tube and used as the plasmid standard series. All samples were analyzed three times by the RT-qPCR assay, and the concentrations of the target DNA detected were expressed as the mean log_10_ of the bacterial genome copy number per milligram of cecal material.

### Statistical Analysis

The data of the laying performance, egg quality, histomorphological parameters, and cecum content of salmonella copies were analyzed by means of one-way ANOVA using the SPSS 22.0 software (SPSS Inc., Chicago, IL, United States). Statistical significance was declared at *P* < 0.05 and trended at *P* < 0.1. The data were expressed as mean ± standard error of mean (SEM), and graphs were generated by the GraphPad Prism software v 8.0 (GraphPad Software, Inc., San Diego, USA).

All statistical analyses were performed in the R environment (version: V3.6.0, http://www.r-project.org/). The alpha diversity indices, including the Sobs index, Ace index, Chao index, and Shannon index, were determined by sampling-based ASV analysis and presented by the observed ASV, which was calculated using the MOTHUR program (version v.1.30.1). Principal coordinates analysis (PCoA) was conducted by the R package (http://www.R-project.org/) to display microbiome beta diversity. The Bray–Curtis metric distances, unweighted Unifrac distances, and weighted Unifrac distances were calculated with the phyloseq package. Adonis (999 permutations) was used to evaluate the effect of *E. faecium* supplementation on the bacterial community structure using the vegan package in R. The differences in the abundance and enrichment of bacterial genera were examined using Kruskal–Wallis tests, and ternary plots were created using the “vcd” package and visualized by the “ggplot2” package in R. The predominance of bacterial communities between groups was analyzed by the linear discriminant analysis (LDA; LDA score > 2.0) effect size method. Based on the normalized relative abundance matrix, the features with significantly different abundances between assigned taxa were determined by the linear discriminant analysis effect size (LEfSe) with the Kruskal–Wallis rank-sum test (*P* < 0.05), and LDA was used to assess the effect size of each feature. The PICRUSt (version 2.0) software was used to predict the Kyoto Encyclopedia of Genes and Genomes (KEGG) ortholog functional profiles of bacterial communities in rhizospheres and roots using the 16S rRNA sequences.

## Results

### *E. faecium* Alleviated the Laying Performance and Egg Quality Damage of *S. Enteritidis*-Challenged Laying Hens

During the post-salmonella challenge period (from day 21 to day 42), compared with the CON group, the egg production of SCON hens had a decreasing trend (0.05 < *P* < 0.1, **Table 1**). Importantly, the level of egg production was significantly higher (*P* < 0.05) from SEF hens than in SCON hens (**Table 1**). Moreover, compared to the CON group, the yolk color was significantly decreased (*P* < 0.05) in the EF group and the shell thickness was decreased (*P* < 0.05) in the SCON group (**Table 2**). Further, the level of shell thickness in the SEF hens was significantly higher (*P* < 0.05) than in SCON hens (**Table 2**). Also, there was no significant difference (*P* > 0.05) in the laying performance, including the egg weight, feed intake, egg mass, and feed efficiency, and in the egg quality, including the yolk weight, albumen weight, eggshell weight, eggshell strength, and Haugh unit of these hens among the treatment groups (**Tables 1**, **2**).

**Table 1:** Effect of dietary supplementation with *E. faecium* on laying performance of hens challenged with *S. Enteritidis*.

Item	CON	EF	SCON	SEF	*P*-value
Egg production, %	86.56 ± 4.10^a,b^	90.15 ± 6.80^a,b^	80.43 ± 11.26^b^	91.16 ± 2.40^a^	<0.05
Egg weight, g/hen	61.27 ± 2.21	60.78 ± 2.27	61.44 ± 0.76	61.09 ± 2.29	0.635
Egg mass, g/d/hen	53.04 ± 3.28	54.80 ± 4.61	49.39 ± 6.65	55.67 ± 1.59	0.055
Feed intake, g/d/hen	108.74 ± 4.09	111.42 ± 13.06	103.93 ± 4.67	110.72 ± 2.51	0.156
Feed efficiency, g egg/g feed	2.05 ± 0.11	2.04 ± 0.19	2.13 ± 0.21	1.99 ± 0.06	0.223

^a,b^Means with different superscripts within a column differ significantly (P < 0.05). Each mean represents five replicates, with 20 hens per replicate.

**Table 2:** Effect of dietary supplement with *E. faecium* on egg quality of layers challenged with *S. Enteritidis*.

Item	CON	EF	SCON	SEF	*P*-value
Egg weight, g	58.14 ± 0.74	60.65 ± 2.01	59.39 ± 1.09	59.60 ± 2.08	0.271
Yolk weight, g	15.39 ± 0.42	15.43 ± 0.39	15.15 ± 0.58	15.38 ± 0.30	0.176
Albumen weight, g	34.58 ± 0.48	37.16 ± 1.94	36.38 ± 0.63	36.16 ± 1.69	0.293
Eggshell weight, g	8.16 ± 0.33	8.06 ± 0.12	7.85 ± 0.16	8.06 ± 0.30	0.248
Yolk weight/egg weight	0.26 ± 0.01	0.25 ± 0.01	0.26 ± 0.01	0.26 ± 0.00	0.433
Albumen weight/egg weight	0.59 ± 0.01	0.61 ± 0.01	0.61 ± 0.00	0.61 ± 0.01	0.132
Eggshell weight/egg weight	0.14 ± 0.00	0.13 ± 0.00	0.13 ± 0.00	0.14 ± 0.00	0.404
Yolk color	5.50 ± 0.58^b^	4.75 ± 0.2^2a^	5.29 ± 0.34^a,b^	4.46 ± 0.52^a,b^	<0.05
Eggshell strength, kg/cm^2^	3.61 ± 0.39	3.27 ± 0.47	3.36 ± 0.27	3.71 ± 0.18	0.153
Shell thickness, mm	0.44 ± 0.02^b^	0.43 ± 0.01^b^	0.40 ± 0.02^a^	0.44 ± 0.01^b^	<0.05
Haugh unit	78.80 ± 8.95	82.06 ± 1.31	84.39 ± 1.78	80.58 ± 1.25	0.107

^a,b^Means with different superscripts within a column differ significantly (P < 0.05). Each mean represents five replicates, with 20 hens per replicate.

### *E. faecium* Affected Host Metabolism, Oxidative Stress Parameters, Inflammatory Cytokines, and Immunoglobulin Levels of *S. Enteritidis*-challenged laying hens

**Figure 2:**
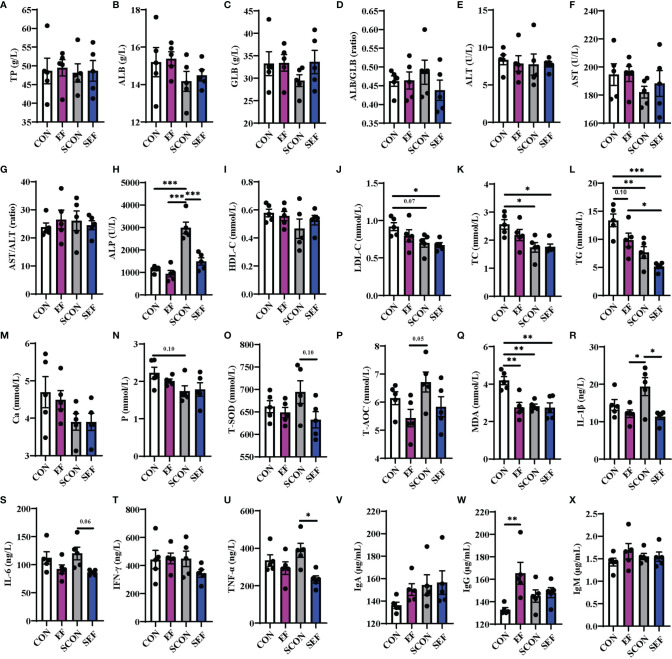
Effect of dietary supplementation with *E. faecium* on serum parameters of hens challenged with *S. Enteritidis*. **(A–H)** TP, ALB, GLB, ALB/GLB, ALT, AST, AST/ALT, and ALP levels in serum samples were measured in different treatments. **(I–N)** HDL-C, LDL-C, TC, TG, Ca, and P levels in serum samples were measured. **(O–X)** T-SOD, T-AOC, MDA, IL-1β, IL-6, IFN-γ, TNF-α, IgA, and IgM levels in serum samples were measured among these groups. Asterisks denote significant differences (*p ≤ 0.05, **p ≤ 0.01, ***p ≤ 0.001), *n* = five per group; data are represented as mean ± SEM.

To evaluate the effects of dietary *E. faecium* supplementation on the host health of laying hens, the protein metabolism, lipid metabolism, oxidative stress, and inflammatory immune responses were recorded and calculated at different phases (the pre-salmonella challenge period and post-salmonella challenge period). As shown in **Figure 2**, during the post-salmonella challenge period (from day 21 to day 42), *S. Enteritidis* infection increased (*P* < 0.05) the concentration of serum ALP and decreased (*P* < 0.05) the levels of serum LDL-C, TC, TG, P, and MDA (**Figures 2H, J–M, Q**), while *E. faecium* treatment significantly decreased (*P* < 0.05) the levels of ALP, T-SOD, IL-1β, IL-6, and TNF-α (**Figures 2H, O, R, S, U**). Also, on day 42, compared to the CON hens, *E. faecium* supplementation downregulated the concentrations of serum TG and MDA and upregulated the level of IgG in the EF group (**Figure 2W**).

### *E. faecium* Improved Intestinal Histopathologic Changes, Alleviated Intestinal Barrier, and Reduced Intestinal Apoptosis of *S. Enteritidis*-Challenged Laying Hens

**Figure 3:**
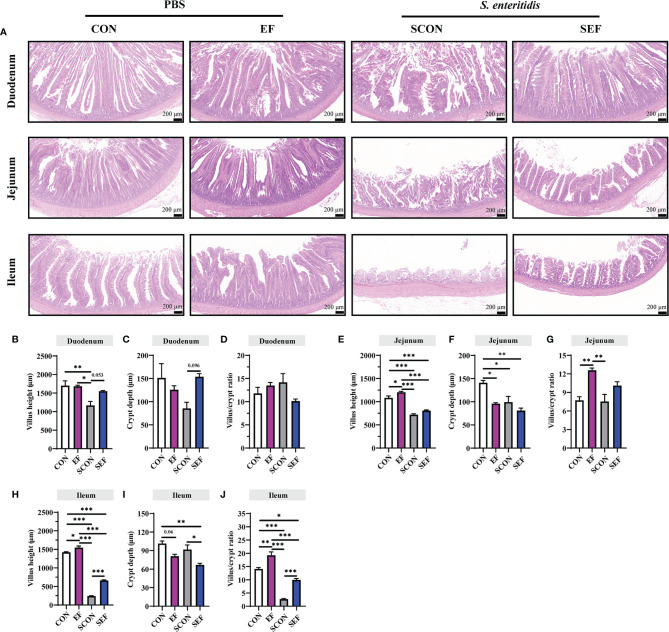
Effect of dietary supplement with *E. faecium* on histomorphological parameters of jejunum and ileum of layers challenged with *S. Enteritidis.* **(A)** Histomorphometric analysis of the Duodenum, Jejunum, and Ileum by Hematoxylin & Eosin (H&E) staining. **(B–J)** The villus height, crypt depth, and villus/crypt ratio shown in the pictures were randomly measured in each sample from each group. Scale bar: 200 μm. Asterisks denote significant differences (*p ≤ 0.05, **p ≤ 0.01, ***p ≤ 0.001), *n* = five per group; data are represented as mean ± SEM.

**Figure 4:**
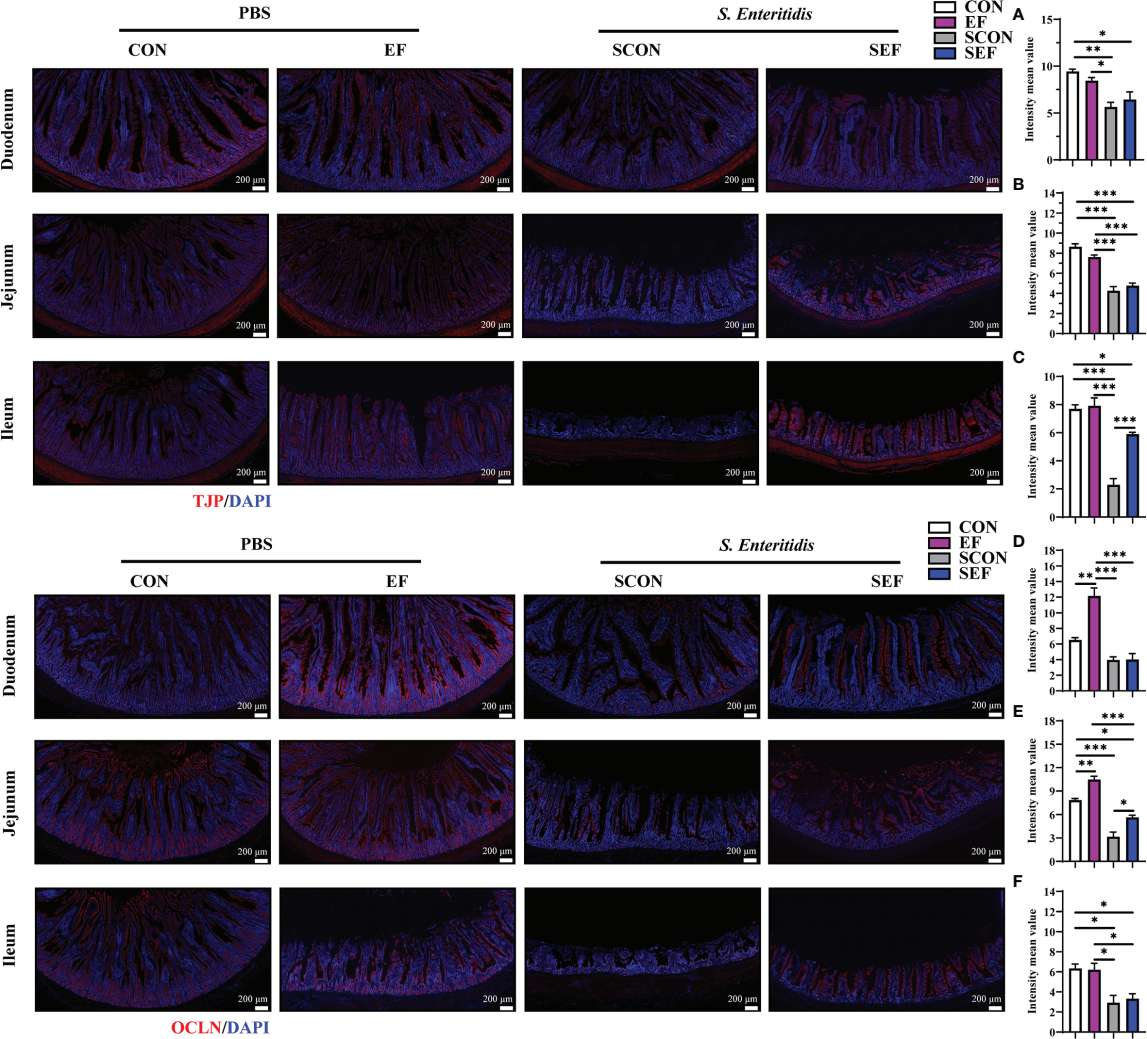
Effect of dietary supplement with *E. faecium* on intestinal epithelial barrier function of layers challenged with *S. Enteritidis.* Representative images of the immunohistochemical staining of TJP in the duodenum **(A)**, Jejunum **(B)**, and ileum **(C)** mucosa. The positive cells are stained red. Representative images of the immunohistochemical staining of OCLN in the duodenum **(D)**, Jejunum **(E)**, and ileum **(F)** mucosa. The positive cells are stained red. Scale bar: 200 μm. Asterisks denote significant differences (*p ≤ 0.05, **p ≤ 0.01, ***p ≤ 0.001), *n* = five per group; data are represented as mean ± SEM.

**Figure 5:**
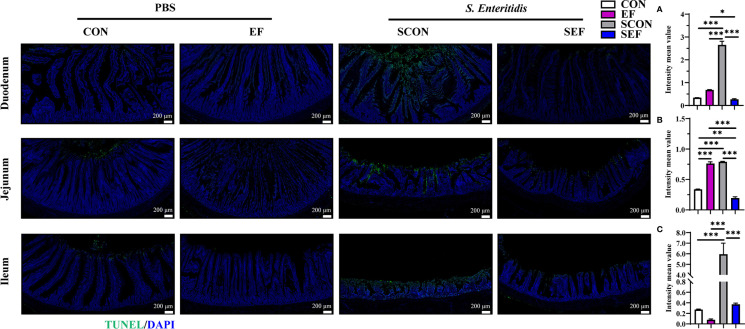
Effect of dietary supplement with *E. faecium* on intestinal epithelial cell apoptosis of layers challenged with *S. Enteritidis.* Representative images of the immunohistochemical staining of TUNEL in the duodenum **(A)**, jejunum **(B)**, and ileum **(C)** mucosa. Scale bar: 200 μm. Asterisks denote significant differences (*p ≤ 0.05, **p ≤ 0.01, ***p ≤ 0.001), *n* = five per group; data are represented as mean ± SEM.

The intestinal morphology of the duodenum, jejunum, and ileum was displayed in **Figure 2**. In our study, obvious hemorrhagic spots were observed (*P* < 0.05) in both jejunum and ileum parts after *S. Enteritidis* challenge. Hematoxylin–eosin (H&E) staining revealed that the intestinal epithelial villi and crypt in the segments of duodenum, jejunum, and ileum were severely damaged (*P* < 0.05) in response to *S. Enteritidis*, as evidenced by the broken villi structure and crypt atrophy (**Figure 3**). However, *E. faecium* administration decreased (*P* < 0.05) the degree of intestinal injury, and reduced the CD, and increased the villus-to-crypt ratio (VCR) in the duodenum, jejunum, and ileum (**Figures 3B–J**). Besides, the salmonella infection significantly decreased (*P* < 0.05) the expression of intestinal barrier-related proteins, such as ZO-1 (TJP1) and OCLN-1 in the duodenum, jejunum, and ileum. However, the expression of TJP1 and OCLN in the SEF group was significantly higher (*P* < 0.05) compared with the SCON group (**Figure 4**). Meanwhile, TUNEL assay results demonstrated that *S. Enteritidis* infection significantly increased the number of positive cells in the duodenum, jejunum, and ileum, while there was a decline following *E. faecium* administration (**Figure 5**).

### *E. faecium* Reshifted the Gut Microbial Community and Structure of S. Enteritidis-Challenged Laying Hens

**Figure 6:**
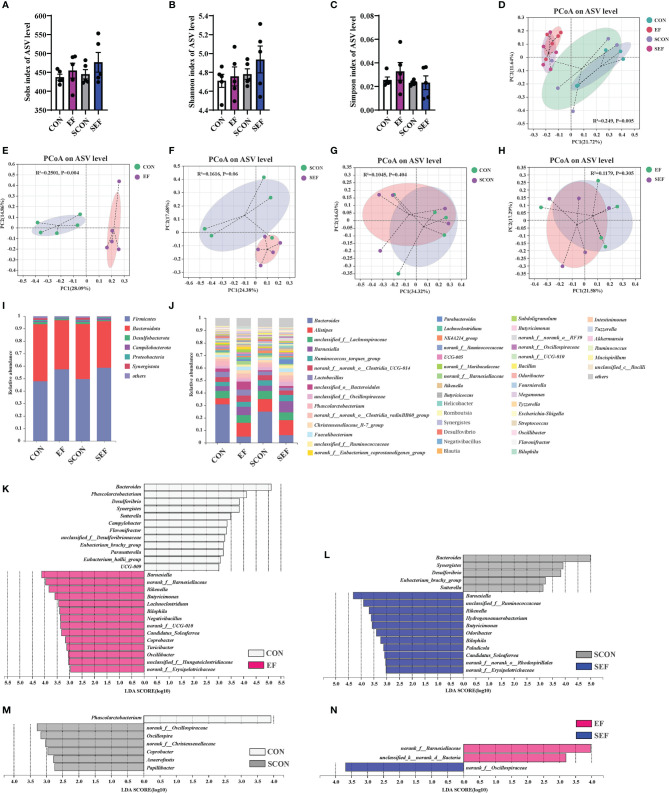
Effect of dietary supplement with *E*. *faecium* on the gut microbial community and structure of cecum in hens. **(A)** Sobs index of ASV level. **(B)** Shannon index of ASV level. **(C)** Simpson index of ASV level. **(D–H)** PCoA plots assessed by Adonis analysis among these treatments. **(I–J)** Shows the relative abundance of phylum and genus in different groups. Linear discriminate analysis effect size (LEfSe) was performed to determine the difference in abundance among these treatments **(K–N)**.

We next used the high-throughput 16S rRNA gene sequencing to determine whether *E. faecium* supplementation affected the gut microbial composition in *S. Enteritidis*-infected hens. V3-V4 16S rRNA gene sequencing was performed on the cecal samples collected from CON, EF, SCON, and SEF hens (n = 5 per group). As shown in **Figures 6A–C**, no significant difference was observed (*P* > 0.05) in the alpha diversity, including the Sobs, Ace, and Chao indices of these hens. A principal coordinate analysis (PCoA) was performed to assess the similarities and differences among groups (**Figures 6D–H**). Our results indicated distinct clusters of the gut microbial composition among these groups. The Adonis analysis based on the Bray–Curtis distance was performed to quantify the differences in species diversity. As shown in **Figures 6D–H**, the data revealed that *E. faecium* administration significantly altered (*P* < 0.05) the β diversity index compared to the control group (EF vs. CON, SEF vs. SCON, respectively). However, these results demonstrated that *S. Enteritidis* infection did not alter the β diversity index of the gut microbiome (**Figures 6G, H**).

As shown in **Figures 6I, J**, phylogenetic analysis revealed the most abundant composition of cecal microbiota at phylum and genera levels among all treatments. At the phylum level, *Firmicutes*, *Bacteroidota*, *Desulfobacterota*, *Campilobacterota*, *Proteobacteria*, and *Synergistota* were dominant (**Figure 6I**). The composition of the gut microbiome at the genera level is shown in **Figure 6J**. The predominant genera were *Bacteroides*, *Alistipes*, *unclassified_f:Lachnospiraceae*, *Barnesiella*, *Ruminococcus_torques_group*, *norank_f:norank_o:Clostridia_UCG-014*, *Lactobacillus*, *unclassified_o:Bacteroidales*, *Phascolarctobacterium*, *Faecalibacterium*, *Parabacteroides*, and so on (**Figrure 6J**). The specific bacterial taxa associated with *E. faecium* and *S. Enteritidis* treatments was identified through the linear discriminant analysis effect size (LEfSe, LDA score > 2.0) analysis. As shown in **Figure 6K** (CON group vs. EF group), there was a significant increase in the relative abundance of *Barnesiella*, *norank_f:Barnesiellaceae*, *Rikenella*, *Butyricimonas*, *Lachnoclostridium*, *Bilophila*, *Negativibacillus*, *Candidatus_Soleaferrea*, *Coprobacter*, *Turicibacter*, and *Oscillibacter* and reduction in the relative abundance of *Bacteroides*, *Phascolarctobacterium*, *Desulfovibrio*, *Synergistes*, *Sutterella*, *Campylobacter*, *Flavonifractor*, and *Parasutterella* in the hens fed with an *E. faecium-*supplemented diet compared to the control hens. As shown in **Figure 6L** (SCON group vs. SEF group), the predominant bacterial strains of SEF were *Barnesiella*, *Rikenella*, *Hydrogenoanaerobacterium*, *Butyricimonas*, *Odoribacter*, *Bilophila*, *Paludicola*, and *Candidatus_Soleaferrea*, while the predominant bacterial strains in the control group (SCON) were *Bacteroides*, *Synergistes*, *Desulfovibrio*, *Eubacterium_brachy_group*, and *Sutterella*. Also, the predominant bacterial strain in the CON group was *Phascolarctobacterium*, while the predominant bacterial strains in the SCON group were *Oscillospira*, *Coprobacter*, *Anaerofustis*, and *Papillibacter* (**Figure 6M**). As shown in **Figure 6N**, the predominant bacterial strains in the EF group were *norank_f:Barnesiellaceae* and *unclassified_k:norank_d:Bacteria*, while the predominant bacterial strain in the SEF group was *norank_f:Oscillospiraceae.*

**Figure 7:**
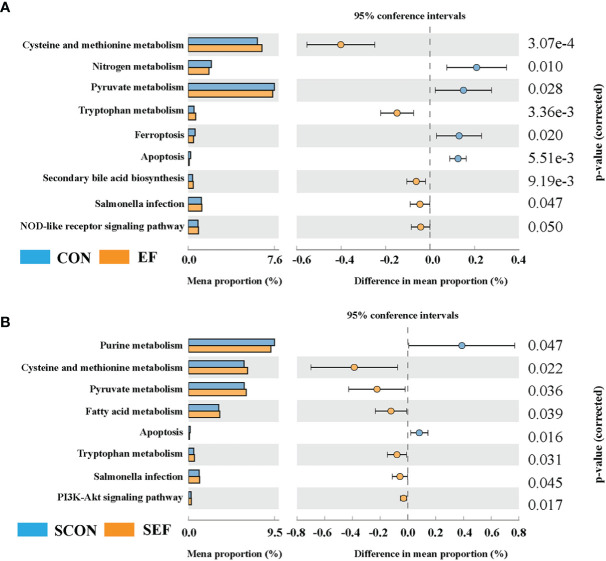
Effect of dietary supplement with *E. faecium* on the gut microbial function of cecum in hens. **(A)** The functional profile of cecum contents between CON and EF groups. **(B)** The functional profile of cecum contents between SCON and SEF groups.

In the present study, Phylogenetic Investigation of Communities by Reconstruction of Unobserved States 2.0 (PICRUSt2) was based on the ASV tree in the Greengenes database to achieve the prediction of the metabolic function of the gut microbiota. As shown in **Figure 7A**, compared to the CON group, the relative abundance of cysteine and methionine metabolism, tryptophan metabolism, secondary bile acid biosynthesis, and the NOD-like receptor signaling pathway were significantly increased in the EF group, while nitrogen metabolism, pyruvate metabolism, ferroptosis, and apoptosis were markedly decreased. Also, our results showed that the abundance of cysteine and methionine metabolism, pyruvate metabolism, fatty acid metabolism, tryptophan metabolism, salmonella infection, and the PI3K-Akt signaling pathway were dramatically increased in the SEF group, while the levels of purine metabolism and apoptosis were decreased compared with the SCON group (**Figure 7B**).

### *E. faecium* Inhibited *S. Enteritidis* Colonization of Laying Hens

**Figure 8:**
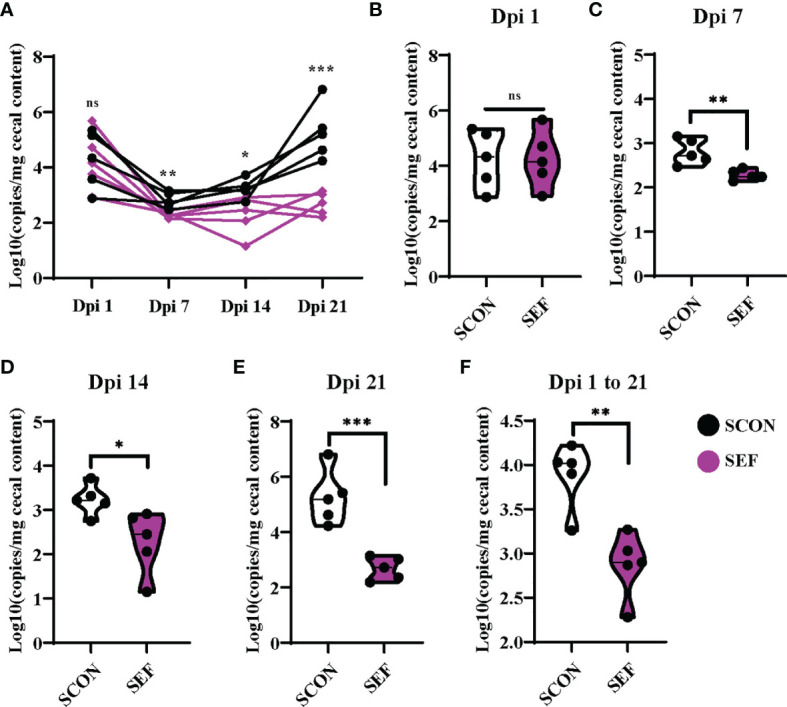
Effect of dietary supplementation with *E. faecium* on salmonella copies in cecum of hens challenged with *S. Enteritidis.* **(A–F)** salmonella copies in the cecum contents determined by RT-qPCR at day 1, 7, 14, 21 post infection (1 Dpi, 7 Dpi, 14 Dpi and 21 Dpi). Asterisks denote significant differences (ns, not significant; *p ≤ 0.05, **p ≤ 0.01, ***p ≤ 0.001), *n* = five per group; data are represented as mean ± SEM.

*S. Enteritidis* copies were detected using the RT-qPCR from cecal samples collected at different time points. Samples were collected from SCON and SEF groups at days 1, 7, 14, and 21 post-infection (1, 7, 14, and 21 dpi). Also, the samples collected from the negative control groups (CON and EF) were negative for *S. Enteritidis* throughout the experiments (data not shown). As shown in **Figures 8A, B**, hens developed infection with *S. Enteritidis* observed in the cecal samples at 1 dpi, while no significant difference was observed between the SCON and SEF groups (*P* > 0.05). Importantly, compared to the SCON, the supplementation of *E. faecium* in the SEF group dramatically decreased (*P* < 0.05) salmonella copies in cecal samples at 7, 14, and 21 dpi (**Figures 8C–E**). These results indicate that *E. faecium* has a strong capacity to inhibit the *S. Enteritidis* colonization of laying hens (**Figure 8F**).

## Discussion

*S. Enteritidis* infection is a serious problem in animal production (e.g., egg products and poultry meat) that ultimately affects the quality and safety of animal-derived foods and human health (37). Numerous previous studies have shown that the *S. Enteritidis* challenge led to a compromised growth performance of poultry. In this study, we found that salmonella exposure resulted in a significant laying performance and egg quality loss, whereas *E. faecium* supplementation reversed these changes. As we have known, the intestine is believed to be the main target organ for salmonella infection. It can destroy the intestinal mucosal barrier, increase gut permeability, and induce intestinal inflammation and bacterial translocation (4). Herein, the *S. Enteritidis* challenge significantly caused host metabolic dysfunction, intestinal histopathologic damage, and intestinal mucosal injuries, whereas *E. faecium* supplementation attenuated these detrimental effects, suggesting the capacity of *E. faecium* to improve the laying performance and support the intestinal homeostasis. In the present study, we have recently identified a crucial role for *E. faecium* in regulating hen cecal commensal microbes that improve beneficial bacteria and inhibit *S. Enteritidis* colonization. These results also revealed that the supplementation of *E. faecium* markedly enriched the cysteine and methionine metabolism, pyruvate metabolism, fatty acid metabolism, tryptophan metabolism, and the PI3K-Akt signaling pathway of hens. Therefore, the supplementation with *E. faecium* has the potential to improve laying hen outcomes.

Previous studies revealed that host cells and organs undergo metabolic reprogramming and activate inflammatory pathways in response to salmonella infection (5, 38). In the present study, *S. Enteritidis* infection significantly increased the serum ALP, whereas *E. faecium* supplementation reversed these alterations. According to a previous study, the abnormalities in liver enzyme levels are frequent during severe intestinal injuries due to *S. Enteritidis* infection (39), which is in line with these findings. However, the level of ALP was decreased in the serum of broilers with fungal infection (40). As reported, oral supplementation with intestinal alkaline phosphatase (IAP) protects mice from infections with *S. Typhimurium* as well as with *Clostridium Difficile* (41). Also, the animals given IAP maintained their weight, had reduced clinical severity and gut inflammation, and showed improved survival (41). Therefore, it remains unclear which dietary supplementation with *E. faecium* directly influences the serum ALP of hens infected with *S. Enteritidis*, the underlying mechanism by which the beneficial functions in *E. faecium* leads to host health needs further study. In laying hens, *S. Enteritidis* has been found in the internal tissues of infected chickens, including the intestine, liver, spleen, lung, ovary, and oviduct (42). Gast et al. reported that salmonella can persist in the liver as a chronic infection for up to 5 weeks after the oral inoculation of hens (43). It is well known that the liver is an important organ for lipid metabolism and the *de novo* fatty acid synthesis (44). Importantly, lipid metabolism in the liver has also been regarded as having an important role in egg maturation and production (44). The induction and maintenance of egg productivity are dependent upon the expression of genes related to lipid metabolism and the generation of egg yolk lipid that is mostly composed of low-density lipoproteins (LDLs) (45). Therefore, *S. Enteritidis* infection could elevate lipid synthesis and reduce lipid transportation in the chicken hepatocytes. It was well known that the concentrations of serum lipid indices are indicative of the host metabolic regulations in a steady state. Of note, as shown in our results, the concentrations of serum TG and LDL-C in the *S. Enteritidis* infection group were decreased compared with the CON group, while no significant differences were observed in the lipid metabolism, including the HDL-C, LDL-C, TC, and TG of these hens (SCON vs. SEF groups). In line with these results, a study revealed that the supplementation of *E. faecium* had no effect on serum lipid parameters, including HDL-C, LDL-C, TG, TC, and other lipid indices of broilers (46). Also, a previous study reported that the administration of *E. faecium* did not affect the serum TG and TC of broilers (47). In brief, it appeared that the serum protein and lipid metabolic parameter response observed in the current study was similar to those of poultry models in previous studies. Therefore, the administration of *E. faecium* did not affect the lipid metabolism of hens, which is likely due to the unchanged lipogenic and lipolytic enzyme activities in the tissues.

The host immune system also activates inflammatory pathways in response to infection with salmonella (48). It was well known that salmonella infection increases susceptibility to intestinal inflammation and contributes to intestinal or systemic inflammation (35, 49). Also, the dysfunction of the host immune system caused by infection is the key factor for salmonella-induced inflammatory injuries (49). Therefore, when the intestinal barrier function is destroyed upon salmonella infection, these pro-inflammatory cytokines, including IL-1β, IL-6, IL-8, IFN, and TNF-α, are activated (5, 50). A previous study reported that a number of cytokines, including IL-1β and TNF-α, are produced during the initial salmonella infection (51). In the current study, no significant difference was observed in the systemic inflammation, including the IL-1β, IL-6, IFN-γ, and TNF-α of these hens (CON and SCON groups), while the pro-inflammatory cytokines IL-1β, IL-6, and TNF-α in the SEF hens were markedly decreased and the IgG was increased than those in the SCON hens. According to previous studies, these cytokines have all been demonstrated to be important for salmonella infection and clearance (52). In line with the outcome, previous studies have demonstrated that some lactic acid bacteria are capable of activating immune responses against pathogen infection and then alleviating host inflammation (53). Importantly, *E. faecium* is one of the first batch of probiotics approved by the European Union and Food and Drug Administration for animal feed (54). As reported that the treatment of intestinal porcine enterocyte cell line (IPEC) cell lines with *E. faecium* HDRsEf1 was found to be effective against pathogen infection (*E. coli*, ETEC) (55). Similarly, numerous studies reported that *E. faecium* showed many beneficial functions, including inhibiting pathogen adhesion and infection, enhancing the anti-inflammatory effect, as well as promoting immune system development (30, 47, 56, 57). These results suggested that *E. faecium* might suppress salmonella-induced systemic inflammation by reducing pro-inflammatory cytokines.

It is well known that gut integrity is a prerequisite for maintaining the host and intestinal homeostasis (58, 59). According to previous studies, the intestinal mucosal barrier comprises connecting epithelial cells that are overlaid by a host-secreted mucous layer and serve as the first line of defense against pathogens and potentially harmful commensal bacteria (60–63). However, the impaired intestinal barrier functions caused by pathogen infection compromises the immune tolerance of the intestines and causes systemic inflammatory responses, which aggravate the systemic immune response and host body damage (64). As we have known, salmonella invades and destroys the intestinal epithelial cells and then crosses the intestinal epithelial barrier to cause intestinal and even systemic inflammation (65, 66). In the current study, significant hemorrhagic spots were observed in the duodenum, jejunum, and ileum on the dpi 42. Also, during the post-salmonella challenge period, the intestinal epithelial villi and crypt in the segments of duodenum, jejunum, and ileum were significantly damaged. For example, the broken villi structure and crypt atrophy were observed, which are consistent with the results from previous reports. Interestingly, the supplementation of *E. faecium* significantly increased the VH and VCR and decreased CD in the duodenum, jejunum, and ileum of hens, and ultimately effectively alleviated the damages of *S. Enteritidis* infection. As previous reported, high VCR is widely regarded as a good biomarker of intestinal mucosal turnover and is related to strong digestion and absorption capacity (67). Similarly, Zhang et al., reported that *E. faecium* YQH2 effectively reduced the colonization of *S.* Typhimurium, which may be attributed to the alleviation of intestinal barrier function damage (66). Meanwhile, according to previous studies, salmonella infection has been shown to regulate certain tight junction proteins, which ultimately promotes the translocation of the bacteria through the intestinal epithelial cell monolayer (68). Also, the manipulation of tight junction proteins serves to damage the intestinal epithelial barrier by increasing its permeability, thereby allowing salmonella to more effectively invade the basolateral side of the epithelial cell monolayer (48). Upon infection with salmonella, OCLN becomes dephosporylated and subsequently removed from epithelial tight junctions (68). In addition, ZO-1 (TJP) is recruited from the cytosol to the membrane, suggesting that salmonella changes the intracellular distribution of this tight junction protein (68). In line with previous studies, *S. Enteritidis* infection significantly downregulated TJP and OCLN protein expression in the small intestinal tissues, exacerbating systemic inflammation in hens. Interestingly, after *E. faecium* supplementation was given to the hens, the improvement expression levels of TJP and OCLN suggested that *E. faecium* restored the intestinal mucosal barrier functions and intestinal health. A previous study reported that *E. faecium* YQH2 improved the intestinal mucosal damage caused by salmonella in chicken (66), which was consistent with the outcome. Also, *E. faecium* YQH2 significantly stimulated the Wnt/β-catenin pathway to promote the repair of intestinal epithelial cells and reduced the intestinal inflammation level (66). Therefore, through the present study, these results indicated that the integrity of intestinal barrier functions in hens infected with *S. Enteritidis* was dramatically destroyed, while *E. faecium* supplementation resulted in the improvement of intestinal or systemic inflammation.

Accumulating evidence has shown that the gut microbiome is consistent with host physiological states, and intestinal physiological structure and function (69–72), and has been regarded as a potential nutritional intervention for the improvement of several intestinal diseases, especially pathogen infection (59, 64, 73). The gut microbiota is increasingly recognized for playing a critical role in human or animal health and disease, especially pathogenic infections, such as salmonella (74). In the present study, both salmonella infection and *E. faecium* supplementation affected the gut microbial alpha- and beta-diversity parameters. Similarly, previous studies indicated that the exposure of chickens to salmonella influences and shapes their gut microbial community and structure (75). As shown in a previous study, salmonella can multiply rapidly and destroy the gut microbiome of young chicks (76). In addition, the salmonella-associated alteration of the gut microbiota could be a result of either pathogen-commensal microbiota interaction or host intestinal mucosal immune responses to the pathogen or even a combination of both (75, 77). As previously reported, the supplementation of *E. faecium* significantly altered the gut microbiota composition of broilers and enriched the relative abundance of short-chain fatty acid-producing microbes (78). Also, *E. faecium* is a natural commensal bacteria of the poultry gastrointestinal tract and is commercially used as a probiotic in poultry diets (47), previous studies reported that numerous types of probiotic (e.g., *Bacillus subtilis*, *Lactobacillus*, and *E. faecium*) supplementation can alter the community and structure of the gut microbiota and improve its diversity (11, 79–81). Based on the PCoA and LEfSe analyses, the gut microbial composition and function are altered in hens, followed with *E. faecium* supplementation. In our study, for both non-infected and infected hens, the relative abundance of *Barnesiella*, *Butyricimonas*, and *Bilophila* were markedly enriched in the supplementation of *E. faecium*, while the abundance of *Bacteroides* was increased in the control hens (CON and SCON groups). As we have known, even though some strains of *Bacteroides* have an anti-inflammatory property, toxigenic *Bacteroides fragilis* induces intestinal inflammation and can cause intestinal diseases and colon cancer (82). For example *Bacteroides*, especially *Gallibacterium*, is an indigenous bacterial pathogen in chicken and one of the major pathogens causing reproductive tract disorders in laying hens (83). As this study reported, these results revealed that orally administered *E. faecium* significantly decreased the relative abundance of *Bacteroides* and then substantially inhibited the intestinal injuries in hens challenged with *S. Enteritidis*, which was similar to the previous result to some extent (84). Earlier studies found that certain *E. faecium* spp. can produce folate, an essential vitamin, which is needed by the body for cell metabolism, cell division, and the synthesis of vitamins and amino acids (54, 85). Also, animal research results indicated that *E. faecium* spp. may boost immune cell function, improve the regulation of cell proliferation, and elevate fat-burning capacity (30, 77, 86). Therefore, *E. faecium* has been confirmed to use some functional compounds as nutritional and immunological substrates to metabolize and regulate beneficial compounds for effectively affecting host and gut microbial metabolism (85, 87, 88). Based on the PICRUSt2 analysis, dietary *E. faecium* may modulate the cysteine and methionine metabolism, Tryptophan metabolism, the NOD-like receptor signaling pathway, and PI3K-Akt signaling pathway and ultimately inhibit salmonella infection by improving the intestinal homeostasis, which was similar to a previous result to some extent. However, the underlying mechanism of effect of *E. faecium* on the bacterial function and host metabolism during salmonella infection needs further research.

## Conclusion

In summary, we found that *E. faecium* supplementation significantly improved the laying performance and egg quality to combat the *S. Enteritidis* challenge. Also, these results demonstrated that *E. faecium* administration dramatically alleviated the intestinal histopathologic damage and improved the intestinal mucosal barrier function injuries caused by *S. Enteritidis* infection. Moreover, the data of 16S rRNA high- throughput sequencing of cecal microflora revealed that *Barnesiella*, *Butyricimonas*, *Bilophila*, and *Candidatus_Soleaferrea* dominated the cecal microflora of hens with *E. faecium* supplementation, which have a higher relative abundance in the known functional genes for cysteine and methionine metabolism, pyruvate metabolism, fatty acid metabolism, tryptophan metabolism, and the PI3K-Akt signaling pathway in *E. faecium*-treated hens from the PICRUSt2 analysis. Very importantly, these results indicate that *E. faecium* has a strong capacity to inhibit the *S. Enteritidis* colonization of laying hens. Therefore, the maintenance of the gut microbial composition protects the intestinal barrier from injury under *S. Enteritidis* infection, as demonstrated by decreased intestinal permeability, enhanced the inhibition of the translocation of bacteria and toxins, and suppressed intestinal inflammation. Our findings provide a scientific foundation for *E. faecium* application in poultry feed in the future.

## Data Availability Statement

The datasets presented in this study can be found in online repositories. The names of the repository/repositories and accession number(s) can be found below: https://www.ncbi.nlm.nih.gov/, PRJNA796704.

## Ethics Statement 

The animal study was reviewed and approved by the institutional Animal Care and Use Committee of China Agricultural University.

## Author Contributions

The authors’ responsibilities were as follows: SH, CJ, JZ, QM, and LZ designed the study. SH, XR, ML, ZL, YG, XW, CJ, and QM conducted the experiments and draft the manuscript. SH and QM polished the manuscript and finished the submission. SH, YG, JZ, CJ, and QM guided to analyze the experimental data. SH, CJ, and QM helped with revisiting and reviewing the manuscript. All authors read and approved the final manuscript.

## Funding

This study was supported by the National Science Foundation of China (grant No.31772621), a Special Fund for China Agricultural Research System program (CARS-40-K08), and the Special Fund from Chinese Universities Scientific Fund (2018TC043).

## Conflict of Interest

The authors declare that the research was conducted in the absence of any commercial or financial relationships that could be construed as a potential conflict of interest.

## Publisher’s Note

All claims expressed in this article are solely those of the authors and do not necessarily represent those of their affiliated organizations, or those of the publisher, the editors and the reviewers. Any product that may be evaluated in this article, or claim that may be made by its manufacturer, is not guaranteed or endorsed by the publisher.
