# Supplementary material for: Intestinal Mucosal Immunity-Mediated Modulation of the Gut Microbiome by Oral Delivery of Enterococcus faecium Against Salmonella Enteritidis Pathogenesis in a Laying Hen Model
Source: Front Immunol. 2022 Mar 15;13:853954. doi: 10.3389/fimmu.2022.853954 (PMC8967290; doi:10.3389/fimmu.2022.853954)
Supplement: Supplementary file 1 [file Table_1.docx]

**Table S1. Ingredient composition and nutrient content of basal diet (%, DM).**

| **Ingredient** | **%** | **Nutrient and energyc**^3^ | |
| --- | --- | --- | --- |
| Corn | 66.45 | Chemical composition | |
| Soybean meal | 22.80 | AME, kcal/kg | 2,700 |
| Limestone | 8.20 | CP, % | 15.52 |
| Dicalcium phosphate | 1.70 | Lysine, % | 0.75 |
| DL-methionine | 0.12 | Methionine, % | 0.37 |
| Dicalcium phosphate | 0.30 | Methionine + cysteine, % | 0.64 |
| Choline chloride | 0.10 | Calcium, % | 3.60 |
| Mineral premix^1^ | 0.30 | Total phosphorus, % | 0.65 |
| Vitamin premix^2^ | 0.03 | Available [phosphorus](http://cn.bing.com/dict/search?q=Phosphorus&FORM=BDVSP6&mkt=zh-cn), % | 0.39 |
| Total | 100.00 |  |  |

1 Vitamin premix supplied (per kg of diet): vitamin A, 6,000 IU; vitamin D3, 1,500 IU; vitamin E, 15 IU; vitamin B1, 3mg; vitamin B2, 10.2 mg; folic acid, 0.9 mg; calcium pantothenate, 15 mg; niacin, 45 mg; vitamin B6, 5.4 mg; vitamin B12, 24μg; biotin, 150 μg;

2 Mineral premix provided (per kg of diet): Cu (CuSO4·5H2O), 6.8 mg; Fe (FeSO4·7H2O), 66 mg; Zn (ZnSO4·7H2O), 83 mg; Mn (MnSO4·H2O), 80 mg; I (KI), 1 mg; Se (Na2SeO3), 0.3 mg;

3 Contents of VK, CP and Ca were analyzed. Contents of other nutrients and energy content were calculated based on Feeding Standard of Chickens.
